# Editorial: Exploring mechanisms of cardiac rhythm disturbances using novel computational methods: Prediction, classification, and therapy

**DOI:** 10.3389/fphys.2023.1155857

**Published:** 2023-02-10

**Authors:** Xin Li, Fernando S. Schlindwein, Jichao Zhao, Martin Bishop, G. André Ng

**Affiliations:** ^1^ School of Engineering, University of Leicester, Leicester, United Kingdom; ^2^ Department of Cardiovascular Sciences, University of Leicester, Leicester, United Kingdom; ^3^ National Institute for Health Research Leicester Cardiovascular Biomedical Research Centre, Glenfield Hospital, Leicester, United Kingdom; ^4^ Auckland Bioengineering Institute, University of Auckland, Auckland, New Zealand; ^5^ School of Biomedical Engineering and Imaging Sciences, King’s College London, London, United Kingdom

**Keywords:** atrial fibrillation, sudden cardiac death, computational methods, machine learning, cardiac arrythmia

## Introduction

Cardiac rhythm disturbances, including arrhythmias and sudden cardiac death (SCD), represent a major worldwide public health problem, accounting for 15%–20% of all deaths (Mehra, 2007; Srinivasan and Schilling, 2018). The electrophysiological mechanisms underlying certain cardiac arrhythmias and SCD are not completely understood. There is still strong debate whether rhythm disturbances such as atrial and ventricular fibrillation are caused entirely by disorganized rhythms, sustained by multiple wavefronts, or if they are caused by organized drivers with subsequent wave breaks and fibrillatory conduction (Nattel, 2003; Nash et al., 2006).

Computational methods for prediction, classification and therapy of cardiac arrhythmias and SCD are of great interest to improve the clinical outcomes of these disorders. However, considerable challenges persist that limit the efficacy and cost-effectiveness of available methodologies. It is therefore vital to develop computational tools to help better understand the underlying mechanisms and improve effectiveness and efficacy of current therapies.

Recent advances in computational power and applications in bioinspired systems including machine learning, big data and statistical mathematics, allow new and more complex architectures with great potential to outperform traditional methods. Novel computational methods applied in electro-anatomic mapping, non-invasive imaging, cardiac clinical and optical mapping, and biophysical computational models will help to describe the mechanisms causing the arrhythmias. A Research Topic compiling these novel computational methods in complex cardiac arrhythmias and SCD may significantly contribute to shed light on clinical applications in prediction, classification and therapy, providing unique and critical importance for management of these significant public health issues.

This Research Topic includes 17 original papers focusing on technological challenges and breakthroughs for mechanisms of cardiac rhythm disturbances using novel computational methods: prediction, classification, and therapy. The papers were co-authored by 149 authors from various science backgrounds, emphasising the importance of interdisciplinary research, particularly by young researchers, in advancing novel computational methods in cardiac research.

### Non-invasive detection, stratification and machine learning

The utilization of machine learning (ML) approaches in the analysis of electrocardiogram (ECG) signals has been proposed as a means of improving the early detection of atrial fibrillation (AF) (Hannun et al., 2019). Studies have demonstrated that these ML algorithms, such as deep learning, decision trees, and support vector machines, possess high accuracy in identifying patterns associated with AF within ECG data (Murat et al., 2021). Additionally, these techniques can also be utilized to predict the likelihood of future events, thereby aiding in risk stratification (Raghunath et al., 2021; Barker et al., 2022). Early detection of AF is crucial for the timely initiation of therapy, as untreated AF can lead to serious complications, including stroke. On this theme, Tseng and Noseworthy reviewed the recent advancements in the use of machine learning techniques in the prediction and screening of atrial fibrillation. They highlighted the effectiveness of various ML algorithms in detecting and predicting AF from ECG signals and how other data such as heart rate variability can also be used for the same purpose. For instance, Valiaho et al. developed a new method for detecting AF that utilizes photoplethysmography (PPG) data. This technique can be integrated into PPG wristbands for AF detection. Mazumder et al. proposed a deep neural architecture for classifying shockable rhythms such as ventricular fibrillation (VF) and ventricular tachycardia (VT) versus other types of non-shockable rhythms. They evaluated their proposed architecture on two open access ECG databases and discovered that the classification accuracy achieved is in compliance with American Heart Association standards for wearable cardioverter defibrillator (WCD). This computational model can also be used for the design and development of personalized WCD vests based on the subject’s specific anatomy and pathology, apart from acting as a device validation test-bed. Accurate detection of ectopic beats is a crucial step in ECG processing, Liu et al. developed a beat-by-beat arrhythmia detection method using weakly supervised deep learning. The model was trained to detect ventricular ectopic beats (VEBs) and supraventricular ectopic beats (SVEBs) on five large, coarsely-annotated datasets. The framework has potential applications in both clinical settings and telehealth. For better treatment planning, Li et al. developed a new ECG classification framework that can differentiate between different levels of organization of fibrillation. This method can non-invasively distinguish AF/VF of different global organization levels from the ECG alone. This framework may be useful in guiding patient selection and mechanism-directed tailored treatment strategies. Improved accuracy of detection and stratification of cardiac complexities can be achieved by utilising a deeper understanding of features obtained from ECG time series data; Li et al. conducted a retrospective study on 1024 consecutive patients who underwent cardiac surgery. They described that certain ECG biomarkers, such as the J wave, T peak to end (Tpe) greater than 112.5 ms, and the amplitude of SV1+RV5 (Sokolow Lyon index) greater than 35 mm, were strong predictors of postoperative ventricular arrhythmias (POVAs). For better utilising non-invasive ECG in clinical pathway, Roudijk et al. conducted the first in-human comparison of non-invasive intracardiac electrocardiogram (iECG) and invasive local activation time (LAT) maps on both the endocardial and epicardial surface during sinus rhythm. They found that iECG and LAT-maps showed improved agreement, but there was considerable absolute difference and moderate correlation coefficient. The study concluded that non-invasive iECG still requires further refinements to facilitate clinical implementation and risk stratification.

### Catheter ablation in atrial fibrillation

Atrial arrhythmias including AF are commonly treated with catheter ablation when medication fails to maintain a patient in normal rhythm. Characterising the atrial electrical activity and structure in patients may be a key step toward a successful intervention. To determine the arrhythmogenic substrate, this stage largely depends on diverse approaches for mapping electrical activity and tissue properties. In this issue, Liao et al. developed a new deep learning (DL) model that can classify the focal source of human AF with high accuracy. The model is trained on raw unipolar electrogram (EGM) data and can automate the process of identifying the focal source (FaST) sites of AF. FaST sites were determined based on data from a single recording location, rather than the activation pattern obtained from a multi-electrode array. The clinical significance of this method is yet to be determined. Roney et al. developed a new time-averaged wavefront analysis method, which showed that there are preferential pathways of activation during AF. They also proposed a new index that measures the propagation of activation waves from the pulmonary vein antra into the atrial body. This index was significantly higher in patients who responded to pulmonary vein isolation (PVI) treatment compared to those who did not respond.

The dominant frequency (DF) of atrial electrograms during AF reflects the local activation rate of the atria. The highest DF sites may play a key role in the maintenance of AF. From a study on 40 patients who underwent a step-CA (stepwise cavotricuspid isthmus ablation) for persistent atrial fibrillation (persAF), Pithon et al. reported that high baseline DF values are predictive of unfavourable ablation outcomes. They also found that a reduction in LAA DF early in the ablation process following PVI is associated with the termination of AF (atrial fibrillation) and maintenance of sinus rhythm in the long term. Chu et al. conducted a prospective AF ablation study using simultaneous whole-chamber non-contact mapping of the highest dominant frequency (HDF). They concluded that targeting dynamic HDF sites is feasible and can be effective, but it lacks specificity in identifying relevant persistent atrial fibrillation (persAF) substrate. They also found that spectral organization may have an adjunctive role in preventing unnecessary substrate ablation. Additionally, they concluded that dynamic HDF sites are not associated with observable rotational activity on isopotential mapping, but epi-endocardial breakthroughs could be contributory. Hwang et al. used realistic computational modelling to study 25 AF patients. They studied that the reduction of DF (dominant frequency) with antiarrhythmic drugs (AADs) is more prominent in the PVs (pulmonary veins) and during a high Smax (maximum spatial dispersion of DF) condition, which results in termination or fragmentation of AF. Additionally, they found that a lower DF and spatially unstable (higher DF-COV) condition also leads to AF termination or fragmentation.

Innovative computational methods are critical components in the robust capture of advanced features. Mase et al. introduced a novel methodology for the characterization of wave propagation and the identification of focal drivers in AF, which is based on the reconstruction of CV vector fields and the application of divergence analysis. They stated that divergence analysis was effective in identifying focal drivers in a complex simulated AF pattern. They also applied this method to human AF mapping data and discovered that it consistently detected focal activation in the pulmonary veins and left atrial appendage area. These results suggest the potential of divergence analysis in combination with multipolar mapping to identify critical sites for AF. Siles-Paredes et al. developed a Circle Method for robust estimation of local conduction velocity high-density maps from optical mapping data. This method aims to provide a more accurate representation of the electrical activity in the heart enables quantitatively predictive studies of how local CV changes affect heart electrophysiology.

Computer models can aid in the discovery of underlying mechanisms of complex cardiac arrythmias. Lyon et al. adapted a computer model of mouse ventricular electrophysiology using experimental data and found that beta-adrenergic stimulation and connexin43 hemichannel-mediated calcium entry contribute to the generation of delayed-afterdepolarizations upon loss of plakophilin-2 function. This work provides insights into potential future antiarrhythmic strategies in arrhythmogenic cardiomyopathy due to plakophilin-2 loss-of-function. Jin et al. developed a realistic computational model of AF and uncovered that circumferential PVI was effective in reducing the DF of AF, increasing its spatial heterogeneity in areas outside of the pulmonary veins, and providing better anti-AF effects than ablation in areas outside of the pulmonary veins or the use of additional flecainide in conditions where gaps existed in PVI.

Additionally, Luo et al. explored the electro-characteristics of myocardial pouches and the relationship between steam pops (SPs), pouches, and impedance. They reported that appropriate delta impedance cutoff settings (percentage of delta impedance (PDI): 15%; delta time: 3 s) can reduce the frequency of SPs and improve the safety of radiofrequency ablation (RFA).

## Conclusion

Cardiac arrhythmias and sudden cardiac death are a major public health problem and the mechanisms underlying these disorders are not fully understood. Computational methods, such as machine learning, big data, and statistical mathematics, can help to improve the effectiveness and efficacy of current therapies by helping better understanding of the underlying mechanisms. Recent advances in these areas, along with other technologies such as electro-anatomic mapping and non-invasive imaging (Salinet et al., 2021), have the potential to significantly improve the management and treatment of these disorders. The future outlook for the field of Electrophysiology (EP) is very promising with many new and innovative advancements expected to emerge in the coming years. With the growth of data analytics, machine learning, and artificial intelligence, the ability to analyse and understand complex ECG data will increase dramatically. Additionally, new technologies such as wearable ECG monitors, non-invasive EP procedures, and real-time monitoring systems are expected to become increasingly prevalent. These advances will improve the accuracy of arrhythmia detection and stratification, leading to more effective and personalized treatments for cardiac patients. Additionally, the development of new drugs and ablation techniques are also expected to play a critical role in the future of EP. Overall, the next decade promises to be an exciting and transformative period for the EP field, and researchers and practitioners alike are looking forward to the many new innovations and advancements that are yet to come. We are pleased to present a Research Topic of studies describing recent investigations into the mechanisms of cardiac rhythm disturbances using novel computational methods for prediction, classification, and therapy. We hope that scientists, engineers, clinicians, and patients interested in the Research Topic will find this overview of basic, clinical, and translational research trends inspiring and encouraging in the development of novel computational methods in cardiac research.
